# Clinical Study of 100 Cases of Dengue at St. Thomas, V. I.

**Published:** 1919

**Authors:** 


**Clinical Study of 100 Cases of Dengue at St. Thomas, V.I.**
By Lieut. F. F. Lane. *U. S. Naval Med. Bulletin*, October 1918.

In November 1917, an epidemic of dengue appeared among Americans who had been on the island three to eight months. Over 200 cases were reported, 140 of which were treated at the Naval Hospital. One hundred records that were carefully kept are the basis of this article.

Dengue is not common in the Virgin Islands ; occasional cases are known among children.

From a study of the 100 cases, the author concludes :

1. The immediate source of the infection was unknown and could only be guessed. The important feature was the agent carrying the infection. The blame can be placed entirely and without hesitation on the *Stegomyia calopus*, practically the only mosquito seen and inhabitating the localities where the disease was most prevalent, and the only place where the mosquito bite seemed to do any damage. The Stegomyia family have been suspected before, but never so definitely as in these cases.

2. The mild character of the bone pains was characteristic.

3. Interesting and previously not definitely reported involvement of the lymph glands, in many cases quite severe, treated with ice. In all 62.7 per cent. of 75 cases free from venereal infection developed this; all large superficial glands throughout the body affected; no breaking down or suppuration noted.

4. Epistaxis occurred in only 10 per cent., but severe and difficult to control.

5. Pain on forced motion of the eyeballs, evidently due to muscular attachments rather than to muscles themselves, elicited only on extreme motion.

6. Very peculiar and sometimes startling symptom of hyperesthesia of an intense variety, noted when the shoulder was accidentally touched by the examiner.

7. Vasomotor phenomena, causing cyanosis and coldness of the arms, hands, lower legs and feet, occurred from sixth to eighth day in about 17 per cent. of cases.

8. Disappearance of the eruption was followed in about 24 hours by quite severe and long-continued desquamation, accompanied by intense itching at times, a sensation as of swelling of the hands and feet, and a continual and profuse sweating of these parts.

9. Blood specimens did not show on the average the very low leucocyte count and the discrepancy in the differential count usually acredited to dengue, yet these findings were sufficiently marked in a number of cases to accord with other descriptions. These blood findings depend largely on the severity of the attack.

10. A disturbing feature in the convalescence from the patient's standpoint was the inability to relish food due to a bad metallic taste, although the appetite was good.

11. All ordinary work could be performed without difficulty after recovery, but the least extra strain brought on intense weakness and fatigue which it took some days, and in many cases, weeks to overcome.

12. Immunity in most cases seems to hold after one attack, but about 5 to 6 per cent. had a recurrence three weeks to several years after the original attacks.

Treatment was entirely symptomatic; patient was given a bath, a purge (usually magnesium sulphate), and put to bed. Ice cap used when headache was severe. As a rule 5 grain doses of aspirin t.i.d.

or q. d. relieve pain and fever, inducing a mild sweat. Water given freely; diet usually soft. When temperature dropped to normal, patient was allowed to do about as he pleased, eat what he wished, and be in or out of bed according to his inclination — this satisfied the patient's mental unrest and had no harmful effects. All cases kept under mosquito bars during the infective stage, supposed to be five days.

The epidemic faded out and in the dry season dengue was practically absent; with the return of the rainy season, new cases developed. Better control of the mosquitoes and their breeding places, and screening are necessary before epidemics can be prevented.

